# Efficacy and safety of stereotactic body radiation therapy combined with hepatic arterial infusion chemotherapy for hepatocellular carcinoma with portal vein tumor thrombosis:a multicenter propensity score matching study

**DOI:** 10.3389/fonc.2026.1768607

**Published:** 2026-02-25

**Authors:** Fengtao Zhang, Biao Liu, Xiang Zheng, Pingkang Chen, Qiming Wei, Liangjie Li, Sheng Zhong, Haiming Zhang

**Affiliations:** 1 Vascular Interventional Surgery, Shenzhen Nanshan People’s Hospital, Shenzhen, Guangdong, China; 2 Department of Interventional Vascular Surgery Room, The First Affiliated Hospital of Jinan University, Guangzhou, Guangdong, China; 3 Department of Interventional Therapy, Zhuhai People’s Hospital(Zhuhai Clinical Medical College of Jinan University), Zhuhai, Guangdong, China; 4 The Radiology Department, The First Affiliated Hospital of Guangzhou Medical University, Guangzhou, Guangdong, China; 5 Department of Interventional, The Second Affiliated Hospital of Guangzhou University of Chinese Medicine, Guangzhou, Guangdong, China; 6 Department of Radiotherapy, The First People’s Hospital of Kashi Prefecture, Kashi, China; 7 Department of Tumor and Vascellum Intervention, DongGuan Tungwah Hospital, DongGuan, Guangdong, China; 8 Department of Radiology, The First Affiliated Hospital of Guangdong Pharmaceutical University, Guangzhou, Guangdong, China

**Keywords:** hepatic arterial infusion chemotherapy, hepatocellular carcinoma, portal vein tumor thrombosis, propensity score matching, stereotactic body radiation therapy

## Abstract

**Background:**

Portal vein tumor thrombosis (PVTT) is among the foremost co-morbidities of hepatocellular carcinoma (HCC), characterized by an inexorably grim prognosis and a paucity of viable therapeutic modalities. Regrettably, the conventional standard sorafenib for HCC patients afflicted with portal vein involvement has yielded suboptimal outcomes. Currently, hepatic arterial infusion chemotherapy (HAIC) and stereotactic body radiation therapy(SBRT) have emerged as promising strategies, particularly in advanced HCC cases complicated by portal vein involvement, showcasing notable survival advantages. Currently, there is no research of HAIC combined with SBRT for HCC patients with PVTT. In this study, we embarked on a multicenter retrospective investigation to elucidate the enhanced survival benefits of this combined modality for HCC patients with portal vein involvement.

**Methods:**

Retrospectively collected clinical data from three medical centers in China on patients with HCC and combined PVTT received HAIC combined with SBRT or HAIC alone between September 2019 and June 2022 were analyzed. 1:1 Propensity Score Matching(PSM) was employed to balance baseline differences between the groups. Survival benefits were compared across cohorts using the Kaplan-Meier method, with subgroup analysis conducted to further elucidate outcomes. Univariate and multivariate analyses based on the COX proportional hazards regression model were performed to identify risk factors associated with survival prognosis. Safety was assessed by comparing adverse event rates between the groups.

**Results:**

In this study, a total of 253 HCC patients with portal vein invasion were included, of whom 79 underwent HAIC combined with SBRT(HAIC-SBRT group) and 174 received HAIC alone(HAIC group). After matching, a total of 146 patients(73 per group) were analyzed. In the matched cohort, the HAIC-SBRT regimen demonstrated significantly superior clinical outcomes compared with HAIC alone. Median overall survival (mOS, 24.5 versus 12.2 months; HR: 0.50, 95% CI: 0.31–0.79; P < 0.001) and median progression-free survival (mPFS, 10.3 versus 6.9 months; HR: 0.67, 95% CI: 0.45–0.99; P = 0.010) were significantly prolonged. The combination therapy also exhibited markedly improved tumor response, achieving superior objective response rates(ORR) for both intrahepatic lesions (61.6% versus 16.4%, P < 0.001) and PVTT (67.1% versus 13.7%, P < 0.001). No statistically significant differences were observed between the two groups in the incidence of grade 1–2 or grade 3–4 adverse events (all P > 0.05).

**Conclusion:**

For HCC with portal vein involvement, the combination of HAIC and SBRT is a more promising and tolerable treatment option compared to HAIC alone.

## 1 Introduction

Hepatocellular carcinoma (HCC) is a highly heterogeneous and aggressive malignant tumor of the liver, ranking among the leading causes of cancer-related deaths worldwide, with its occurrence often accompanied by intricate comorbidities, leading to a poor prognosis (1–3). Portal vein tumor thrombus (PVTT) stands as one of the major severe complications of HCC, categorizing the condition as advanced upon its occurrence, with an incidence rate ranging from approximately 44% to 62% (4, 5). Given that the portal vein serves as the primary blood supply pathway for the liver, its involvement signifies a significantly elevated risk of intrahepatic metastasis and is a primary manifestation of high tumor burden. Furthermore, portal hypertension induced by portal vein involvement leads to impaired liver function or even liver failure, which are the primary factors limiting the efficacy of treatments and restricting treatment options for HCC patients (6). These factors contribute to the extremely poor prognosis for this specific population. Reports indicate that the median overall survival (mOS) of HCC patients with PVTT receiving placebo treatment is merely 2.7 to 4.0 months (7). Currently, systemic therapy is endorsed by the BCLC guidelines as the first-line treatment option for HCC with PVTT. Unfortunately, as one of the standard first-line options, oral sorafenib treatment also has limited efficacy, extending survival by less than 2 months (8).

As a form of arterial local treatment, transarterial chemoembolization (TACE) is widely recognized as the standard treatment regimen for intermediate-stage and certain advanced HCC. However, there remains significant debate regarding the applicability of TACE for HCC patients with portal vein involvement, primarily due to concerns over portal hypertension induced by the tumor thrombus and the risk of liver failure following hepatic arterial embolization (9). Hepatic artery infusion chemotherapy (HAIC) stands as a distinct localized chemotherapy approach from conventional systemic chemotherapy, involving the prepositioning of microcatheters in the hepatic artery for the sustained slow infusion of chemotherapeutic agents, which not only achieves direct tumor cytotoxicity through local chemotherapy but also avoids the first-pass effect of the liver, thereby enhancing the efficacy of the chemotherapy. Preliminary reports from several phase III clinical trials indicate that for unresectable HCC, HAIC treatment significantly improves patient survival outcomes and achieves tumor shrinkage compared to traditional TACE and sorafenib, particularly in cases of high tumor burden with portal vein involvement (10–12). Additionally, with the advent of the combination therapy era for HCC, comprehensive treatments based on HAIC are increasingly being employed for HCC with portal vein involvement (13). A recent randomized controlled trial reported mOS and objective response rates (ORR) of an encouraging 16.3 months and 41.0%, respectively, in patients with main portal vein tumor thrombus treated with HAIC combined with sorafenib (14).

In addition to systemic therapy, local radiotherapy has also demonstrated favorable management capabilities for HCC with tumor thrombosis. Traditional radiotherapy has limited application in HCC due to the low tolerance of high tumor burden HCC to radiation and the risk of radiation-induced liver disease (15). Advances in radiotherapy, such as three-dimensional conformal radiotherapy (3D-CRT) and image-guided radiotherapy, allow for higher radiation doses to be delivered to tumor tissues outside of normal liver tissue. Stereotactic body radiation therapy (SBRT) is a 3D-CRT technique designed to optimize precise target dose delivery and protect normal tissues while reducing the number of chemotherapy sessions to enhance targeted antitumor effects. Increasing evidence now shows that SBRT can significantly improve the prognosis of HCC patients with portal vein involvement (16, 17). A recent randomized trial showed that SBRT treatment showed higher local antioxidant activity compared to Transarterial Chemoembolization with Drug Eluting Beads (DEB-TACE) (18). Another previous studies have reported that the combination of SBRT and TACE can achieve adequate thrombus shrinkage and facilitate conversion (19). Building on these findings and the advantages of HAIC over TACE alone or in combination, the combination of HAIC and SBRT is a promising treatment approach. Currently, there is a lack of systematic real-world evidence regarding the HAIC combined with SBRT’ regimen for the treatment of patients with HCC with PVTT.

Despite the potential benefits of the combined HAIC and SBRT treatment regimen, this approach remains an unexplored area in the management of HCC with PVTT. Therefore, we conducted a multicenter retrospective study to investigate the safety and efficacy of the combined HAIC and SBRT treatment regimen.

## 2 Materials and methods

### 2.1 Study design and patients

The multicenter retrospective study reviewed and analyzed clinical data from patients with HCC and concomitant PVTT treated with HAIC combined with SBRT (HAIC-SBRT group) or HAIC alone (HAIC group) from three medical centers in China between September 2019 and June 2022. The Institutional Ethics Committee waived the need for informed consent due to the retrospective nature of this study. The participating centers in this study are listed in **Supplementary Table 1**. All written treatment informed consent was obtained prior to HAIC treatment.

All HCC diagnoses were confirmed according to the criteria of the American Association for the Study of Liver Diseases (AASLD) or the European Association for the Study of the Liver (EASL) or by liver biopsy (20, 21). Inclusion criteria were as follows: (1) age 18–65 years; (2) Eastern Cooperative Oncology Group performance status (ECOG PS) score 0-1; (3) clear radiological evidence of portal vein tumor thrombosis; (4) liver function ALBI grade 2 or Child-Pugh A-B grade; (5) adequate hematologic, with leukocyte count < 3.0 × 10^9/^L, neutrophil count < 1.5 × 10^9/^L, platelet count < 75 × 10^9/^L, and hemoglobin < 85 g/L. **Exclusion criteria included:** (1) missing clinical data; (2) loss to follow-up for more than 6 months; (3) presence of other malignancies; (4) prior treatment other than HAIC.

### 2.2 HAIC procedures

All hepatic artery catheterization procedures were performed by at least two experienced interventional radiologists under digital subtraction angiography (DSA). The process was as follows: After local anesthesia, the femoral artery was punctured using the modified Seldinger technique, and a 5F vascular sheath was placed. A 5F Yashiro catheter (Terumo, Tokyo, Japan) was inserted through the sheath into the hepatic artery, and arteriography was performed to identify the tumor-supplying arteries and their branches. Subsequently, a microcatheter (ASAHI, Tokyo, Japan) was placed in the target tumor-feeding artery and secured externally. Following the hepatic artery catheterization, the mFOLFOX6 regimen was administered, consisting of: Oxaliplatin at a dose of 85 mg/m^2^ intravenously over 2 hours on day 1; Leucovorin at a dose of 400 mg/m^2^ intra-arterially over 2 hours on day 1; Fluorouracil at a dose of 400 mg/m^2^ given by intra-arterial injection, followed by a continuous infusion of 2400 mg/m^2^ over 46 hours, all delivered through the microcatheter. Dose adjustments were made in cases of persistent or severe treatment-related adverse reactions, with treatment resumed once the patient’s condition stabilized. The HAIC procedures were repeated every four weeks, the total number of HAIC treatment cycles did not exceed 6 sessions. Post-treatment, a full abdominal enhanced CT scan was conducted every eight weeks for treatment evaluation.

### 2.3 Stereotactic body radiation therapy procedures

SBRT simulation was performed within 1–2 days after the completion of the first HAIC cycle (continuous infusion). The entire SBRT course, with a total dose of 36–40 Gy delivered in 5 fractions on an every-other-day schedule, was executed and completed within 2–4 weeks after the first HAIC session. Subsequent HAIC cycles continued at the predetermined frequency (once every 4 weeks). SBRT was performed as follows: Four-dimensional computed tomography(4D CT) was employed in the treatment planning process to account for respiratory motion. The gross tumor volume (GTV), delineated through dynamic enhanced CT or MRI, encompassed both the portal vein tumor thrombus (PVTT) and the contiguous primary hepatic lesion. In cases where the primary disease was large, multicenter, or diffuse, rendering the entire tumor volume extensive and precluding the sparing of adequate normal liver from high radiation doses, only the PVTT was considered as the GTV. The internal target volume (ITV) was established to accommodate the range and position of the tumor throughout the breathing cycle, and the planning target volume (PTV) was generated with a uniform expansion of the ITV by 3 to 5 mm. Manual adjustments were made to the PTV to minimize bowel overlap when necessary. The initial prescribed dose to the PTV was 40 Gy administered in five fractions, utilizing 6 MV X-rays via a linear accelerator weekly (Varian Medical Systems). In instances where the PTV was in close proximity to the bowel or sufficiently large to pose challenges in meeting the dose-volume constraints of the organs at risk, the dose was adjusted to a range of 36 to 39 Gy across 5 to 6 fractions.

The dose-volume constraints for organs at risk were as follows: for the liver, the constraint was set at ≥ 700 mL of uninvolved liver (liver minus GTV) receiving < 15 Gy, or alternatively, ensuring that the percentage of normal liver volume receiving more than 15 Gy (V_15_) did not exceed one-third of the total normal liver volume (V_total_); the maximum dose to 1 mL of the stomach, small bowel, or duodenum was restricted to < 25 Gy; for the spinal cord, the maximum dose to 1 mL was limited to < 15 Gy; and for the kidneys, V_15_ was required to be less than one-third of V_total_.

### 2.4 Study endpoint

The primary endpoint of this study is overall survival (OS), calculated from the initial definitive diagnosis of HCC to the patient’s death or the last follow-up. Tumor response is evaluated according to the modified Response Evaluation Criteria in Solid Tumors 1.1 (mRECIST 1.1) criteria, by two radiologists independently. Image-based assessments categorize results into complete response (CR), partial response (PR), stable disease (SD), and progressive disease (PD). The secondary endpoint is progression-free survival (PFS), calculated from the initial definitive diagnosis of HCC to the first image-based evaluation as PD according to mRECIST 1.1.The third endpoint is tumor response, including the objective response rate (ORR), the proportion of patients evaluated as CR and PR during imaging follow-up, and the disease control rate (DCR), the proportion of patients evaluated as CR, PR, and SD during imaging follow-up. Adverse events are documented and assessed according to the Common Terminology Criteria for Adverse Events 5.0 (CTCAE 5.0) criteria.

### 2.5 Propensity score matching

Propensity score matching (PSM) with 1:1 ratio were utilized to mitigate baseline disparities, with a tolerance threshold of 0.02 for matching. The covariates incorporated into the balancing process encompassed age, gender, ECOG PS score, comorbidities, HBsAg status, cirrhosis presence, ascites, Child-Pugh classification, ALBI grade, alpha-fetoprotein (AFP) levels, maximum tumor diameter, tumor number, extrahepatic metastasis and HAIC sessions.

### 2.6 Statistical analysis

All statistical analysis was conducted using R software (RStudio version 4.2.2). For continuous variables adhering to a normal distribution, the mean ± standard deviation was used for representation and analyzed via Student’s t-test. For non-normally distributed continuous variables, the median was utilized for representation and analyzed using the Mann-Whitney U test. Categorical variables were expressed as percentages and analyzed using either the chi-square test or Fisher’s exact test. The Kaplan-Meier method was employed to estimate overall survival (OS) and progression-free survival (PFS) curves between groups in both the overall and matched cohorts. Univariate and multivariate analysis based on Cox proportional hazards regression model was applied to identify independent risk factors affecting OS and PFS. Covariates with a P-value < 0.1 in the univariate analysis were collectively included in the multivariate analysis.

A two-tailed P-value < 0.05 was considered statistically significant.

## 3 Results

### 3.1 Patients characteristics

**Figure 1:**
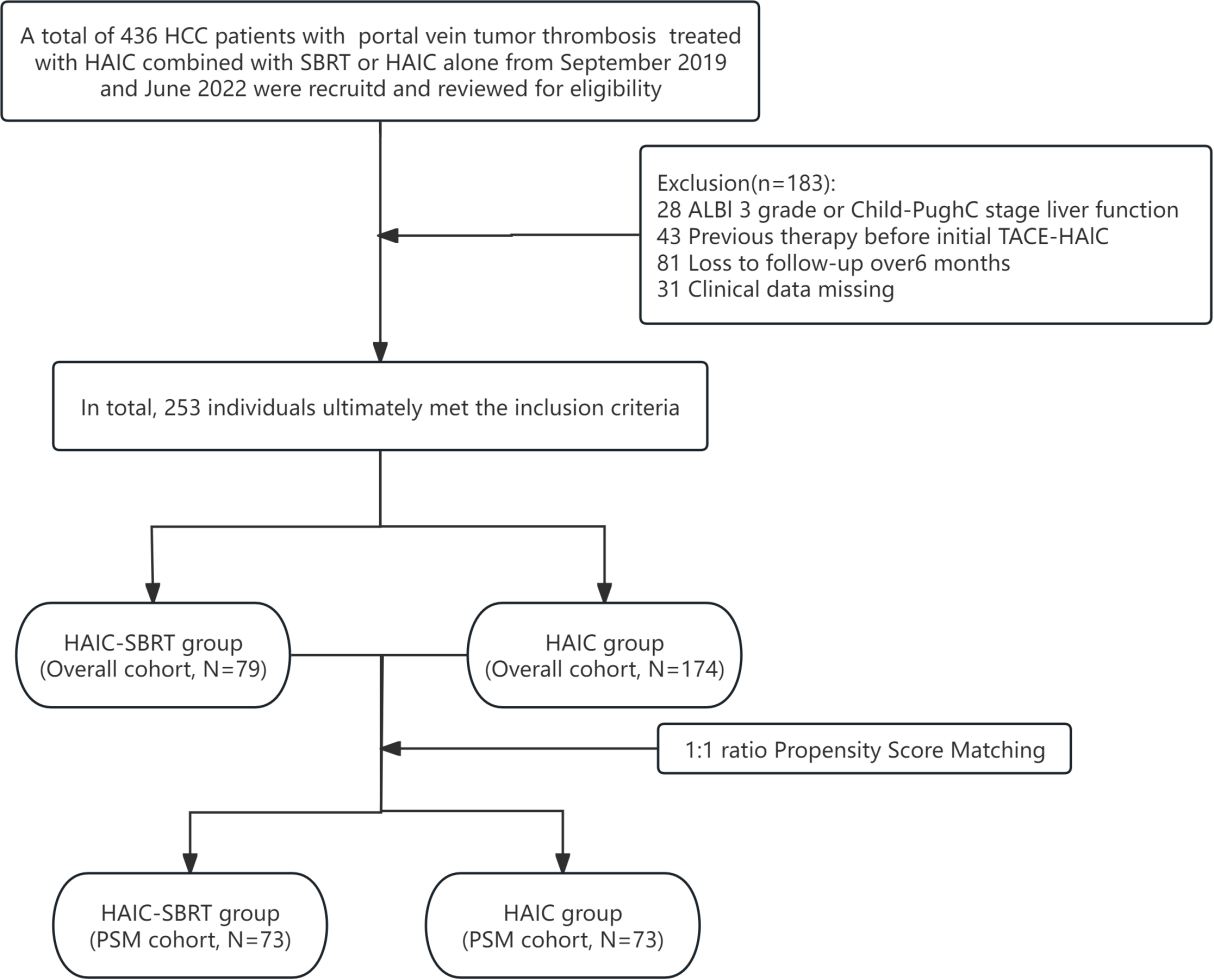
Flowchart of the patients selection process for this study.

After qualification review, a total of 253 HCC patients with PVTT who received HAIC combined with SBRT treatment (n = 79) or HAIC-alone treatment (n = 174) met the inclusion criteria and were enrolled. The enrollment case screening flowchart is shown in **Figure 1**. In the overall cohort, the majority of enrolled patients had general characteristics such as positive HBsAg (93.3%), ECOG PS 0 (91.7%), age ≤ 65 years (91.3%), and male (88.5%). The average age and tumor diameter in the HAIC-SBRT group were 49.1 ± 11.2 years and 10.5 ± 3.5 cm, respectively, while in the HAIC group, they were 51.4 ± 11.6 years and 11.1 ± 3.9 cm, respectively. The HAIC-SBRT group had a significantly higher proportion of patients with ascites and ALBI grade 1. After 1:1 propensity score matching, the above differences were eliminated, and a total of 146 PSM cohort patients were obtained. Baseline conditions before and after propensity matching are shown in **Table 1**.

**Table 1:** Baseline characteristics of the study patients before and after PSM.

Covariates	Entire cohort			PSM cohort		
	HAIC-SBRT (n=79)	HAIC (n=174)	P value	HAIC-SBRT (n=73)	HAIC (n=73)	P value
Gender			0.084			0.172
Male	74 (93.7%)	150 (86.2%)		69 (94.5%)	72 (98.6%)	
Female	5 (6.3%)	24 (13.8%)		4 (5.5%)	1 (1.4%)	
Age	49.1 ± 11.2y	51.4 ± 11.6y	0.368	49.6 ± 10.8y	50.7 ± 11.5y	0.754
≤65y	74 (93.7%)	157 (90.2%)		68 (93.%)	67 (91.8%)	
>65y	5 (6.3%)	17 (9.8%)		5 (6.8%)	6 (8.2%)	
ECOG PS			0.080			0.311
0	76 (96.2%)	156 (89.7%)		70 (95.9%)	72 (98.6%)	
1	3 (3.8%)	18 (10.3%)		3 (4.1%)	1 (1.4%)	
Comorbidities			0.902			0.785
Presence	10 (12.7%)	23 (13.2%)		8 (11.0%)	7 (9.6%)	
Absence	69 (87.3%)	151 (86.8%)		65 (89.0%)	66 (90.4%)	
HBsAg			0.748			0.649
Presence	75 (94.9%)	161 (92.5%)		70 (95.9%)	71 (97.3%)	
Absence	4 (5.1%)	13 (7.5%)		3 (4.1%)	2 (2.7%)	
Cirrhosis			0.269			0.261
Presence	67 (84.8%)	156 (89.7%)		64 (87.7%)	68 (93.2%)	
Absence	12 (15.2%)	18 (10.3%)		9 (12.3%)	5 (6.8%)	
Ascites			0.016			0.754
Presence	6 (7.6%)	34 (19.5%)		6 (8.2%)	5 (6.8%)	
Absence	73 (92.4%)	140 (80.5%)		67 (91.8%)	68 (93.2%)	
Child-Pugh grade			0.251			1.000
A	77 (97.5%)	160 (92.0%)		71 (97.3%)	71 (97.3%)	
B	2 (2.5%)	10 (8.0%)		2 (2.7%)	2 (2.7%)	
ALBI grade			0.030			0.859
1	55 (69.6%)	96 (55.2%)		50 (68.5%)	49 (67.1%)	
2	24 (30.4%)	78 (44.8%)		23 (31.5%)	24 (32.9%)	
AFP			0.273			0.603
≤400ng/L	31 (39.2%)	56 (32.2%)		27 (37.0%)	24 (32.9%)	
>400ng/L	48 (60.8%)	118 (67.8%)		46 (63.0%)	49 (67.1%)	
Tumor size	10.5 ± 3.5cm	11.1 ± 3.9cm	0.093	10.7 ± 4.2cm	11.5 ± 4.2cm	1.000
≤7cm	13 (16.5%)	16 (9.2%)		10 (13.7%)	10 (13.7%)	
>7cm	66 (83.5%)	158 (90.8%)		63 (86.3%)	63 (86.3%)	
Tumor number			0.137			0.300
1-3	34 (43.0%)	58 (33.3%)		29 (39.7%)	23 (31.5%)	
>3	45 (57.0%)	116 (66.7%)		44 (60.3%)	50 (68.5%)	
Metastasis			0.405			0.730
Presence	52 (65.8%)	105 (60.3%)		48 (65.8%)	46 (63.0%)	
Absence	27 (34.2%)	69 (39.7%)		25 (34.2%)	27 (37.0%)	
HAIC sessions			0.521			0.738
1-3	32 (40.5%)	78 (44.8%)		30 (41.1%)	32 (43.8%)	
≥3	47 (59.5%)	96 (55.2%)		43 (58.9%)	41 (56.2%)	

P-value < 0.05 indicated a significant difference.

PSM, Propensity Score Matching; HAIC-SBRT, Hepatic arterial infusion chemotherapy combined with Stereotactic Body Radiation Therapy; HAIC, Hepatic arterial infusion chemotherapy; ECOG PS, Eastern cooperative oncology group performance status; HBsAg, hepatitis B surface antigen; ALBI, albumin-bilirubin; AFP, α-fetoprotein.

### 3.2 Survival outcomes

**Figure 2:**
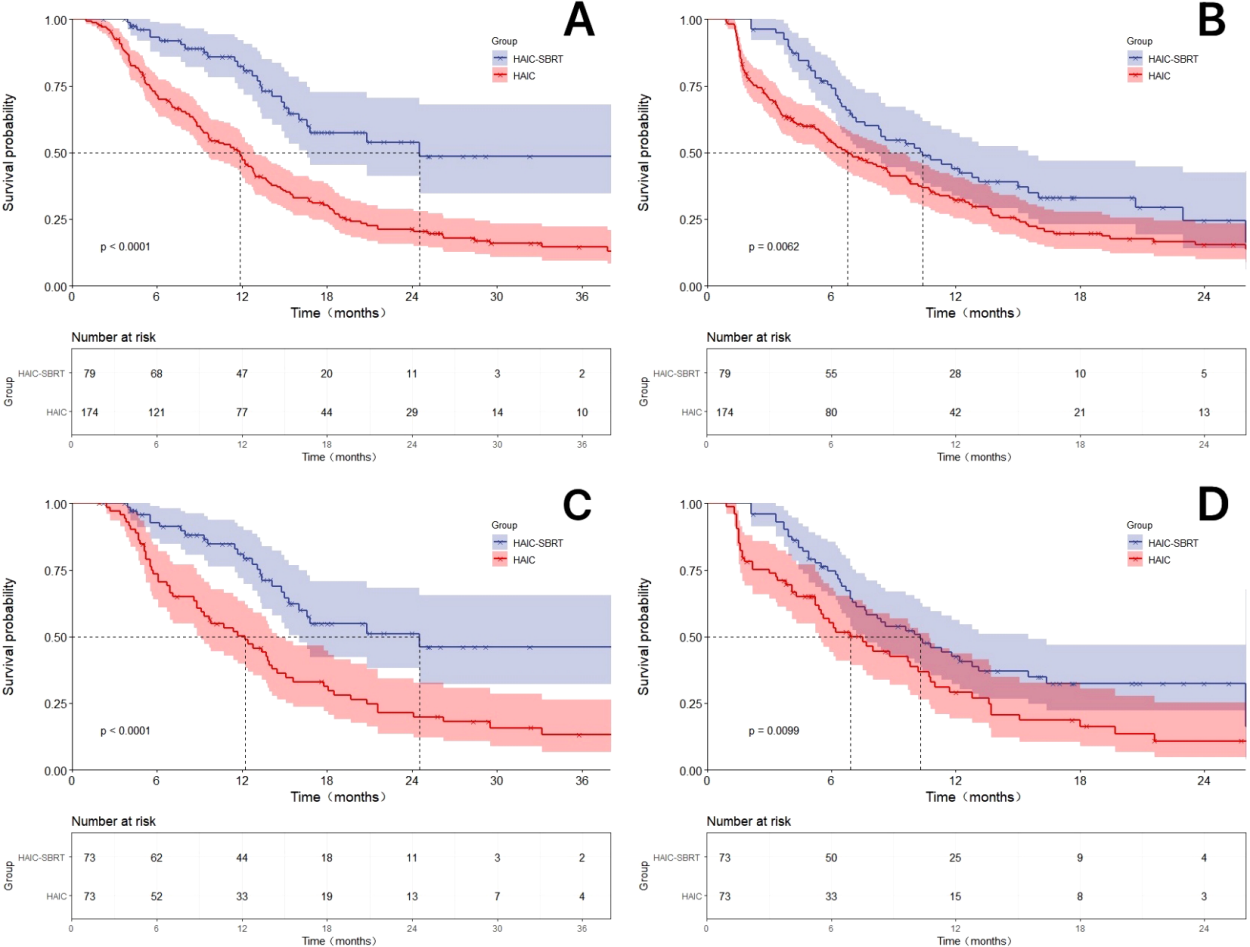
The Kaplan-Meier survival curves by Log-rank test for the HAIC-SBRT group and the HAIC group with or without propensity score matching(PSM) adjustment. **(A)** The Kaplan-Meier curves comparing the overall survival between the HAIC-SBRT group and the HAIC group without PSM-adjusted; **(B)** The Kaplan-Meier curves comparing the progression-free survival between the HAIC-SBRT group and the HAIC group without PSM-adjusted; **(C)** Comparison of PSM-adjusted overall survival between the HAIC-SBRT group and HAIC groups; **(D)** Comparison of PSM-adjusted progression-free survival between the HAIC-SBRT group and HAIC groups.

In the overall cohort, the median OS for the HAIC-SBRT group and the HAIC group was 24.5 months and 11.9 months, respectively (HR: 0.49, 95% CI: 0.32-0.74; P < 0.0001). The median PFS was 10.4 months and 6.8 months, respectively (HR: 0.65, 95% CI: 0.47-0.91; P = 0.0062). After propensity matching to balance differences between groups, the median OS and PFS for the HAIC-SBRT group were 24.5 months and 10.3 months, respectively, both significantly better than the 12.2 months (HR: 0.50, 95% CI: 0.31-0.79; P < 0.0001) and 6.9 months (HR: 0.67, 95% CI: 0.45-0.99; P = 0.010) for the HAIC group. In the matched cohort, the 6, 12, and 24-month OS rates for the HAIC-SBRT group were 84.9%, 60.3%, and 15.1%, and the 6, 12, and 18-month PFS rates were 68.5%, 34.2%, and 12.3%, all significantly higher than the 71.2%, 45.2%, and 7.8% for OS and 45.2%, 20.5%, and 11.0% for PFS in the HAIC group (all P < 0.001). The propensity matching adjusted and unadjusted Kaplan-Meier survival curves were exhibited in **Figure 2**.

In the HAIC-SBRT group, due to varying tumor burdens among patients, the GTV scope was determined through multidisciplinary team(MDT) consensus, selecting either “intrahepatic lesion plus PVTT” or “PVTT only.” This approach aimed to avoid exceeding the tolerance limits of normal liver tissue and prevent radiation-induced liver disease or insufficient preservation of functional liver reserve, which could result from delivering radical radiation doses to all intrahepatic tumor. In this study cohort, among HCC patients in the HAIC-SBRT group, the median OS for those with “intrahepatic tumor-PVTT” and “PVTT-only” as the GTV scope was 20.8 months and 24.5 months(HR: 1.18, 95% CI: 0.55 to 2.54; P = 0.271), respectively. The median PFS was 11.4 months and 9.6 months(HR: 1.19, 95% CI: 0.69 to 2.04; P = 0.102), respectively. No statistically significant differences were observed in either outcome. The Kaplan-Meier curves stratified by GTV scope are presented in **Supplementary Figure 2**.

### 3.3 Tumor response

According to the mRECIST 1.1 criteria, in the overall cohort, the HAIC-SBRT group achieved CR in 1(1.3%) case, PR in 46(58.2%) cases, SD in 23(29.1%) cases, and PD in 9(11.4%) cases, while the HAIC group achieved CR in 1(0.6%) case, PR in 29(16.7%) cases, SD in 88(50.6%) cases, and PD in 56(32.2%) cases (P < 0.001). The ORR and DCR of intrahepatic tumor in the HAIC-SBRT group were 59.5% and 88.6%, respectively, significantly higher than those in the HAIC group, which were 17.2% and 67.8% (both P < 0.001). After propensity matching, the HAIC-SBRT group demonstrated similarly superior anti-tumor efficacy in terms of overall response, ORR, and DCR. In the PSM cohort, the HAIC-SBRT and HAIC-alone groups achieved CR in 1(1.4%) and 1(1.4%) case, PR in 44(60.3%) and 11(15.1%) cases, SD in 20(27.4%) and 42(57.5%) cases, and PD in 8(11.0%) and 19(26.0%) cases, respectively (P < 0.001). The ORR of intrahepatic tumor lesion in the HAIC-SBRT and HAIC-alone groups were 61.6% and 16.4% (P < 0.001), respectively, and the DCR were 89.0% and 74.0% (P = 0.019).In addition, the ORR and DCR of PVTT in the HAIC-SBRT group of PSM cohort were 67.1% and 97.3%, respectively, significantly higher than the 13.7% and 54.8% in the HAIC group (both P < 0.001). The best tumor response before and after propensity matching adjustment is shown in **Table 2**.

**Table 2:** Best tumor response before and after propensity matching adjustment.

Tumor response	Overall cohort			PSM cohort		
	HAIC-SBRT group(n=79)	HAIC group (n=174)	P value	HAIC-SBRT group(n=73)	HAIC group (n=73)	P value
**Best Response**			**< 0.001**			**< 0.001**
Intrahepatic tumor						
CR	1 (1.3%)	1 (0.6%)		1 (1.4%)	1 (1.4%)	
PR	46 (58.2%)	29 (16.7%)		44 (60.3%)	11 (15.1%)	
SD	23 (29.1%)	88 (50.6%)		20 (27.4%)	42 (57.5%)	
PD	9 (11.4%)	56 (32.2%)		8 (11.0%)	19 (26.0%)	
ORR	59.5% (47/79)	17.2% (30/174)	**< 0.001**	61.6% (45/73)	16.4% (12/73)	**< 0.001**
DCR	88.6% (70/79)	67.8% (118/174)	**< 0.001**	89.0% (65/73)	74.0% (54/73)	**0.019**
PVTT						
CR	3 (3.8%)	1 (0.6%)	**< 0.001**	3 (4.1%)	1 (1.4%)	**< 0.001**
PR	48 (60.8%)	22 (12.6%)		46 (63.0%)	9 (12.3%)	
SD	25 (31.6%)	69 (39.6%)		22 (30.1%)	30 (41.1%)	
PD	3 (3.8%)	82 (47.1%)		2 (2.7%)	33 (45.2%)	
ORR	64.6% (51/79)	13.2% (23/174)		67.1% (49/73)	13.7% (10/73)	
DCR	96.2% (76/79)	52.9% (92/174)	**< 0.001**	97.3% (71/73)	54.8% (40/73)	**< 0.001**
**Conversion to resection**	48.1% (38/79)	9.2% (16/174)	**< 0.001**	49.3% (39/73)	9.6% (7/73)	**< 0.001**

PSM, Propensity Score Matching; HAIC-SBRT, Hepatic arterial infusion chemotherapy combined with Stereotactic Body Radiation Therapy; HAIC, Hepatic arterial infusion chemotherapy; CR Complete response, PR, partial response, SD, Stable disease, PD, Progressive disease, ORR, Objective response rate, DCR, Disease control rate, PVTT, protal vein tumorthombsis. Bold indicates statistical significance level at p-value < 0.05.

### 3.4 Univariate and multivariate analysis

Based on the COX proportional hazards regression model, covariates with a P-value < 0.01 in univariate analysis were jointly included in multivariate analysis. Univariate analysis showed that ALBI grade 2, tumor diameter > 7 cm, tumor number > 3, and HAIC-alone treatment were risk factors associated with worse OS, while AFP level > 400 ng/ml, extrahepatic metastasis, and HAIC-alone treatment were risk factors associated with poorer PFS. Multivariate analysis showed that ALBI grade 2, tumor diameter > 7 cm, and HAIC-alone treatment were independent risk factors affecting OS, while AFP level > 400 ng/ml, extrahepatic metastasis, and HAIC-alone treatment were independent risk factors affecting PFS. The univariate and multivariate analyses based on COX regression are shown in **Table 3**.

**Table 3:** Risk factors for overall survival and progression-free survival based on uni- and multivariate analysis.

Factors	Overall survival				Progression-free survival			
	Univariate analysis P value	Multivariate analysis			Univariate analysis P value	Multivariate analysis		
		HR	95%CI	P value		HR	95%CI	P value
Gender	0.997	–	–	–	0.839	–	–	–
Male								
Female								
Age	0.320	–	–	–	0.737	–	–	–
≤65y								
>65y								
ECOG PS	0.107	–	–	–	0.173	–	–	–
0								
1								
Comorbidities	0.989	–	–	–	0.568	–	–	–
Presence								
Absence								
HBsAg	0.942	–	–	–	0.164	–	–	–
Presence								
Absence								
Crrihosis	0.972	–	–	–	0.200	–	–	–
Presence								
Absence								
Ascites	0.212	–	–	–	0.215	–	–	–
Presence								
Absence								
Child-Pugh grade	0.487	–	–	–	0.264	–	–	–
A								
B								
ALBI grade	**0.025**	0.32	0.16-0.59	**0.008**	0.318	–	–	–
1								
2								
AFP	0.484	–	–	–	**0.008**	0.51	0.39-1.08	**0.012**
≤400ng/mL								
>400ng/mL								
Tumor size	**0.053**	0.55	0.38-0.74	**0.033**	0.734	–	–	–
≤7cm								
>7cm								
Tumor number	**0.096**	1.20	0.86-1.87	0.282	0.287	–	–	–
1-3								
>3								
Metastasis	0.165	–	–	–	**0.037**	1.37	1.02-1.84	**0.039**
Presence								
Absence								
Treatment regimen	**< 0.001**	0.39	0.26-0.59	**< 0.001**	**< 0.001**	0.57	0.41-0.79	**0.001**
HAIC-SBRT								
HAIC								

HR, Hazard ratios; CI, Confidence interval; ECOG PS, Eastern cooperative oncology group performance status; HBsAg, Hepatitis B surface antigen; ALBI, Albumin-bilirubin ratio; AFP, α-fetoprotein; HAIC-SBRT, Hepatic arterial infusion chemotherapy combined with Stereotactic Body Radiation Therapy; HAIC, Hepatic arterial infusion chemotherapy. Bold indicates statistical significance level at p-value < 0.1.

### 3.5 Subgroup analysis

**Figure 3:**
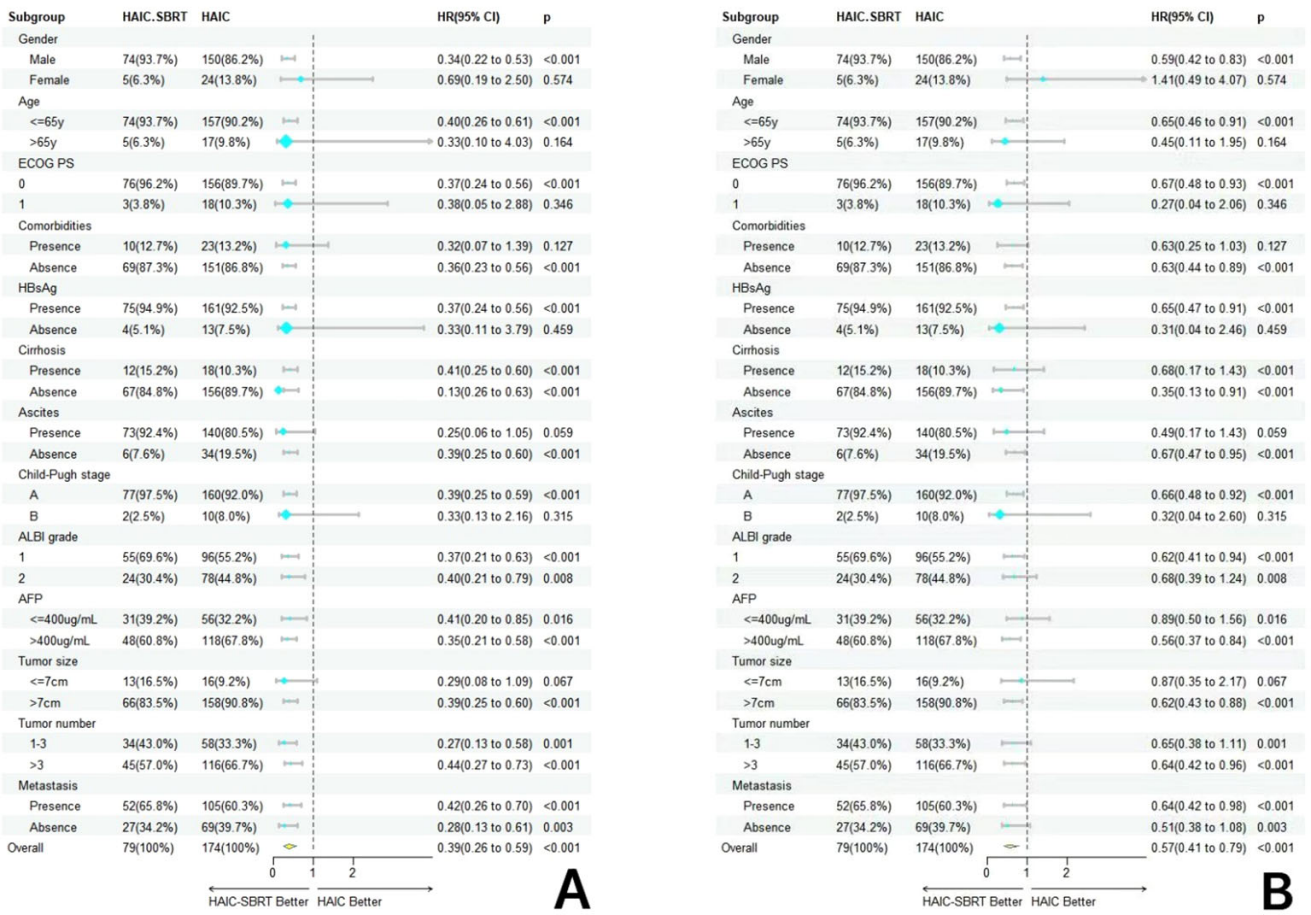
Forest plot based on overall survival **(A)** and progression-free survival **(B)** of each subgroup.

Subgroup analysis revealed that, with the exception of subgroups characterized by female gender, age > 65 years, ECOG PS 0, absence of comorbidities, and HBsAg negativity, the combined HAIC and SBRT regimen exhibited superior overall survival benefits and more effective tumor progression control compared to HAIC treatment alone across all other subgroups. The forest plots illustrating this subgroup analysis are depicted in **Figure 3**.

### 3.6 Safety

During the treatment period, no cases of treatment-related mortality were reported. All treatment-related adverse reactions are detailed in **Table 4**. Abdominal pain, nausea, and elevated AST were the most frequent Grade 1–2 adverse events observed in both the HAIC-SBRT group and the HAIC group. These symptoms were noticeably alleviated or resolved following appropriate symptomatic management. In the HAIC-SBRT group, the prevalent Grade 3–4 adverse reactions included elevated transaminases (AST elevation: 19.0%; ALT elevation: 10.1%), thrombocytopenia (10.1%), and hyperbilirubinemia (10.1%). For the HAIC group, the primary Grade 3–4 adverse reactions were elevated transaminases (AST elevation: 11.6%; ALT elevation: 7.5%), thrombocytopenia (8.0%), and leukopenia (4.6%). After matching, similarly, abdominal pain, nausea, and elevated AST remained the most frequent Grade 1–2 adverse events in both the HAIC-SBRT and HAIC groups. The most common Grade 3–4 adverse reactions in the HAIC-SBRT group included elevated AST (19.2%), elevated ALT (11.1%), hyperbilirubinemia (10.1%), and thrombocytopenia (9.6%). In the HAIC group, the most frequent Grade 3–4 adverse reactions were elevated AST (16.4%), leukopenia (8.2%), thrombocytopenia (6.8%), and hyperbilirubinemia (6.8%). Treatment-related adverse events after matching are presented in **Supplementary Table 2**. Statistical analysis revealed no significant differences in Grade 1–2 or Grade 3–4 treatment-related adverse events before and after PSM between the HAIC-SBRT group and the HAIC group.

**Table 4:** Treatment-related adverse events.

Adverse events	Grade 1/2			Grade 3/4		
	HAIC-SBRT (n=79)	HAIC (n=174)	P value	HAIC-SBRT (n=79)	HAIC (n=174)	P value
Hypertension	11 (13.9%)	19 (10.9%)	0.493	0 (0%)	1 (0.6%)	0.500
Diarrhea	13 (16.5%)	25 (14.4%)	0.667	0 (0%)	0 (0%)	1.000
Nausea	40 (50.6%)	83 (47.7%)	0.665	0 (0%)	0 (0%)	1.000
Vomiting	25 (31.6%)	51 (29.3%)	0.707	1 (1.3%)	1 (0.6%)	0.565
Fever	15 (19.0%)	23 (13.2%)	0.234	4 (5.1%)	3 (1.7%)	0.133
Abdominal pain	41 (51.9%)	79 (45.4%)	0.338	5 (6.3%)	3 (1.7%)	0.121
Neurologic toxicity	14 (17.7%)	31 (17.8%)	0.985	1 (1.3%)	1 (0.6%)	0.565
Hand-foot syndrome	9 (11.4%)	18 (10.3%)	0.803	1 (1.3%)	2 (1.1%)	0.937
Elevated ALT	33 (41.8%)	64 (38.5%)	0.449	8 (10.1%)	13 (7.5%)	0.478
Elevated AST	38 (48.1%)	78 (44.8%)	0.628	15 (19.0%)	28 (16.1%)	0.570
Anemia	34 (43.0%)	72 (41.4%)	0.804	3 (3.8%)	3 (1.7%)	0.275
Leukopenia	15 (19.0%)	29 (16.7%)	0.652	7 (8.9%)	8 (4.6%)	0.183
Neutropenia	9 (11.4%)	15 (8.6%)	0.486	4 (5.1%)	6 (3.4%)	0.541
Thrombocytopenia	23 (29.1%)	46 (26.4%)	0.658	8 (10.1%)	14 (8.0%)	0.586
Hypoalbuminemia	32 (40.5%)	69 (39.7%)	0.898	5 (6.3%)	5 (2.9%)	0.191
Hyperbilirubinemia	20 (25.3%)	42 (24.1%)	0.840	8 (10.1%)	7 (4.0%)	0.106
Elevated creatinine	7 (8.9%)	14 (8.0%)	0.828	0 (0%)	2 (1.1%)	0.339
Proteinuria	5 (6.3%)	8 (4.6%)	0.563	1 (1.3%)	0 (0%)	0.137

HAIC-SBRT, Hepatic arterial infusion chemotherapy combined with Stereotactic Body Radiation Therapy; HAIC, Hepatic arterial infusion chemotherapy; ALT, Alanine aminotransferase; AST, Aspartateaminotransferase.

## 4 Discussion

This study is the first multicenter real-world evidence supporting HAIC combined with SBRT as one of the new treatment options for HCC with PVTT. In this study, the HAIC-SBRT treatment regimen significantly extended OS and PFS for HCC patients with portal vein involvement compared to HAIC alone. In terms of tumor response, the combined therapy exhibited more advantageous anti-tumor effects, with higher ORR and DCR. Additionally, nearly half of the patients treated with HAIC-SBRT achieved downstaging and clinical conversion to surgery. These results suggest that the HAIC-SBRT regimen is superior to HAIC monotherapy in this patient population.

Portal vein involvement is one of the most intractable complications of HCC and one of the primary reasons for exclusion from surgical candidacy, portending a poor prognosis and limited therapeutic options. As the standard treatment for HCC with PVTT, sorafenib alone limited anti-tumor capacity and only achieved a median OS of 5.5 to 7.2 months (11, 22). Previous phase III trials reported that FOLFOX-HAIC was superior to standard sorafenib in extending survival and reducing tumor burden in HCC with PVTT (12). Currently, HAIC has been recommended by the Japan Society of Hepatology (JSH) as one of the preferred treatment options for HCC with macroscopic vascular invasions (MVI) (23). However, due to tumor heterogeneity, monotherapy with HAIC only benefits a small fraction of patients with portal vein involvement (24). Several previous studies have reported satisfactory results with comprehensive treatment regimens based on HAIC. Notably, for the population with HCC and portal vein involvement, radiotherapy appears to play a crucial role in their treatment, with its specific advantage over systemic and transarterial therapies being the ability to exert local effects on tumor thrombosis. SBRT stands out from conventional radiotherapy for its precision, especially its anti-tumor effects on intravascular tumor thrombosis, which play a primary role in intrahepatic and extrahepatic metastasis and directly limit survival prognosis (25). Several previous studies have reported the efficacy of HAIC combined with 3D radiotherapy in treating HCC with portal vein involvement. A recent retrospective study by Chia-Ling Chiang et al. reported the effectiveness of HAIC combined with radiotherapy in controlling disease progression and reducing tumor size, achieving an impressive ORR of 80.0% and a PFS of 9.0 months (26). Kenichiro Kodama et al. reported the efficacy of HAIC combined with radiotherapy in treating patients with main portal vein tumor thrombi, achieving a median OS of 9.9 months, approximately twice that of standard sorafenib therapy (27). Additionally, another previous meta-analysis also reported positive results for HAIC combined with radiotherapy (28). This study is the first to report the application of HAIC combined with SBRT in HCC with PVTT, demonstrating superior outcomes compared to previous HAIC combined with conventional radiotherapy.

The survival benefit of the combined therapeutic regimen in this study can be attributed to the synergistic effect of both treatments. Firstly, mFOLFOX6, which encompasses 5-fluorouracil, calcium folinate, and oxaliplatin, directly targets tumor cells, disrupting their DNA structure and inhibiting their proliferation. Simultaneously, SBRT employs high-dose radiation to directly eradicate tumor cells, complementing the direct tumor cell killing mechanisms of chemotherapy (29, 30). Secondly, chemotherapy alters the tumor microenvironment, driving tumor cells into the more radiosensitive S phase or G2/M phase of the cell cycle, thereby enhancing treatment efficacy (31). Concurrently, SBRT directly reduce tumor volume, diminishing the tumor’s barrier effect on chemotherapy drugs and thereby increasing local drug concentration and strengthening the chemotherapy effect through precise targeting of high-dose radiation (32). Additionally, tumor growth is reliant on the formation of new blood vessels, and chemotherapy can either destroy these vessels or inhibit angiogenic factors, reducing tumor blood supply and lowering tumor resistance to anticancer drugs and radiation (33, 34).

In this study, multivariate analysis demonstrated that ALBI grade, tumor diameter, and the treatment regime were independent risk factors influencing OS. The AFP level, extrahepatic metastasis, and treatment regime were identified as independent risk factors affecting PFS. Compared to the Child-Pugh classification, ALBI grade, which is based on objective criteria, emerged as a more precise method for assessing liver function, and the correlation between baseline liver function and treatment efficacy as well as long-term survival has been substantiated (33, 35). Tumor diameter, on the other hand, serves as a primary reflection of tumor burden. In addition, existing clinical evidence has confirmed the correlation between AFP and the invasiveness of HCC (36, 37), as well as the undeniable fact that extrahepatic metastasis is a major factor in poor prognosis and progression of HCC.

In subgroup analysis, the combined therapy of HAIC and SBRT was found to significantly outperform HAIC alone, with the exception of subgroups defined by female gender, age > 65 years, ECOG Performance Status 0, absence of comorbidities, and HBsAg negativity, where no significant statistical differences were observed. This discrepancy was primarily attributed to the bias in subgroup sample sizes due to the selective enrollment of patients. In terms of safety, liver function impairment and cytopenia were the most common adverse reactions, with no significant statistical differences in the occurrence rates of various adverse reactions between the groups.

Based on our knowledge, this study represents the first report on the efficacy and safety of combining HAIC with SBRT for treating HCC with PVTT and there were still several shortcomings of this study must be acknowledged. First, as a retrospective study, the selection bias in the enrollment of patients was inevitable. Although propensity matching was used to balance baseline differences, the inherent disparities could not be completely eliminated. Second, the relatively small number of enrolled cases led to bias in some subgroup analyses. Therefore, future studies require larger-scale, randomized controlled trials to further validate these findings. Additionally, another primary limitation of this study is its focus on HBV-related HCC. Consequently, our findings may not be fully generalizable to HCC arising from other prevalent etiologies, such as HCV infection, non-alcoholic fatty liver disease (NAFLD) or alcohol-related HCC, particularly in Western populations. Finally, the current study did not include collection of patient quality of life (QoL) data or document combination therapies with immunotherapy, which warrants further investigation in subsequent research.

## 5 Conclusion

In summary, the combined therapy of HAIC and SBRT offers a promising treatment approach by significantly improving survival benefits and achieving liver lesion and tumor thrombus remission thereby reducing tumor burden in HCC patients with PVTT, represents a potentially beneficial option for this patient population, warranting further validation through prospective or randomized studies.

## Data Availability

The raw data supporting the conclusions of this article will be made available by the authors, without undue reservation.
