# Supplementary material for: Efficacy and safety of stereotactic body radiation therapy combined with hepatic arterial infusion chemotherapy for hepatocellular carcinoma with portal vein tumor thrombosis:a multicenter propensity score matching study
Source: Front Oncol. 2026 Feb 25;16:1768607. doi: 10.3389/fonc.2026.1768607 (PMC12975471; doi:10.3389/fonc.2026.1768607)
Supplement: Supplementary file 1 [file DataSheet1.docx]

**Efficacy and Safety of Stereotactic Body Radiation Therapy combined with Hepatic Arterial Infusion Chemotherapy for Hepatocellular Carcinoma with Portal Vein Tumor Thrombosis：A Multicenter Propensity Score Matching Study**

**This supplementary material includes:**

**1. Supplementary Table1.** The data source form multi-center hospitals.

**2. Supplementary Table2.** Treatment-related adverse events in the matched cohort.

**3. Supplementary Figure1.** Center random effects plot.

**4. Supplementary Figure2.** Kaplan-Meier curves stratified by GTV scope in the HAIC-SBRT group.

**Supplementary Table1.** The data source form multi-center hospitals

| **Hospitals’ name** | **HAIC-SBRT group N=79 (%)** | **HAIC group**  **N=174(%)** |
| --- | --- | --- |
| Shenzhen Nanshan People’s Hospital(Affiliated Nanshan Hospital of Shenzhen University) | 41(51.9) | 70(40.2) |
| Zhuhai People's Hospital(Zhuhai Clinical Medical College of Jinan University) | 23(29.1) | 39(22.4) |
| The First Affiliated Hospital of Guangdong Pharmaceutical University | 15(19.0) | 65(37.4) |

**Supplementary Table2.** Treatment-related adverse events in the matched cohort.

| **Adverse events** | **Grade 1/2** | | |  | **Grade 3/4** | | |
| --- | --- | --- | --- | --- | --- | --- | --- |
|  | **HAIC-SBRT**  **(n=73)** | **HAIC**  **(n=73)** | **P value** |  | **HAIC-SBRT**  **(n=73)** | **HAIC**  **(n=73)** | **P value** |
| Hypertension | 9(12.3%) | 10(13.7%) | 0.806 |  | 0(0%) | 1(1.4%) | 0.316 |
| Diarrhea | 13(17.8%) | 10(13.7%) | 0.496 |  | 0(0%) | 0(0%) | 1.000 |
| Nausea | 37(50.7%) | 33(45.2%) | 0.508 |  | 0(0%) | 0(0%) | 1.000 |
| Vomiting | 24(32.9%) | 21(28.8%) | 0.591 |  | 1(1.4%) | 0(0%) | 0.316 |
| Fever | 13(17.8%) | 12(16.4%) | 0.826 |  | 4(5.5%) | 2(2.7%) | 0.404 |
| Abdominal pain | 39(53.4%) | 33(45.2%) | 0.321 |  | 4(5.5%) | 3(4.1%) | 0.698 |
| Neurologic toxicity | 12(16.4%) | 13(17.8%) | 0.826 |  | 1(1.4%) | 1(1.4%) | 1.000 |
| Hand-foot syndrome | 9(12.3%) | 7(9.6%) | 0.596 |  | 1(1.4%) | 1(1.4%) | 1.000 |
| Elevated ALT | 30(41.1%) | 28(38.4%) | 0.735 |  | 8(11.0%) | 5(6.8%) | 0.383 |
| Elevated AST | 36(49.3%) | 32(43.8%) | 0.507 |  | 14(19.2%) | 12(16.4%) | 0.665 |
| Anemia | 32(43.8%) | 34(46.6%) | 0.739 |  | 3(4.1%) | 2(2.7%) | 0.649 |
| Leukopenia | 14(19.2%) | 12(16.4%) | 0.665 |  | 6(8.2%) | 6(8.2%) | 1.000 |
| Neutropenia | 9(12.3%) | 8(11.0%) | 0.796 |  | 4(5.5%) | 2(2.7%) | 0.404 |
| Thrombocytopenia | 22(30.1%) | 19(26.0%) | 0.581 |  | 7(9.6%) | 5(6.8%) | 0.547 |
| Hypoalbuminemia | 30(41.1%) | 28(38.4%) | 0.735 |  | 5(6.3%) | 3(4.1%) | 0.467 |
| Hyperbilirubinemia | 20(27.4%) | 20(27.4%) | 1.000 |  | 8(11.0%) | 5(6.8%) | 0.383 |
| Elevated creatinine | 7(8.9%) | 6(6.8%) | 0.771 |  | 0(0%) | 1(1.4%) | 0.316 |
| Proteinuria | 5(6.3%) | 3(4.1%) | 0.467 |  | 1(1.3%) | 0(0%) | 0.316 |

**Abbreviation:** HAIC-SBRT: Hepatic arterial infusion chemotherapy combined with Stereotactic Body Radiation Therapy; HAIC: Hepatic arterial infusion chemotherapy; ALT: Alanine aminotransferase; AST: Aspartate aminotransferase.

**3. Supplementary Figure1.** Center random effects plot.


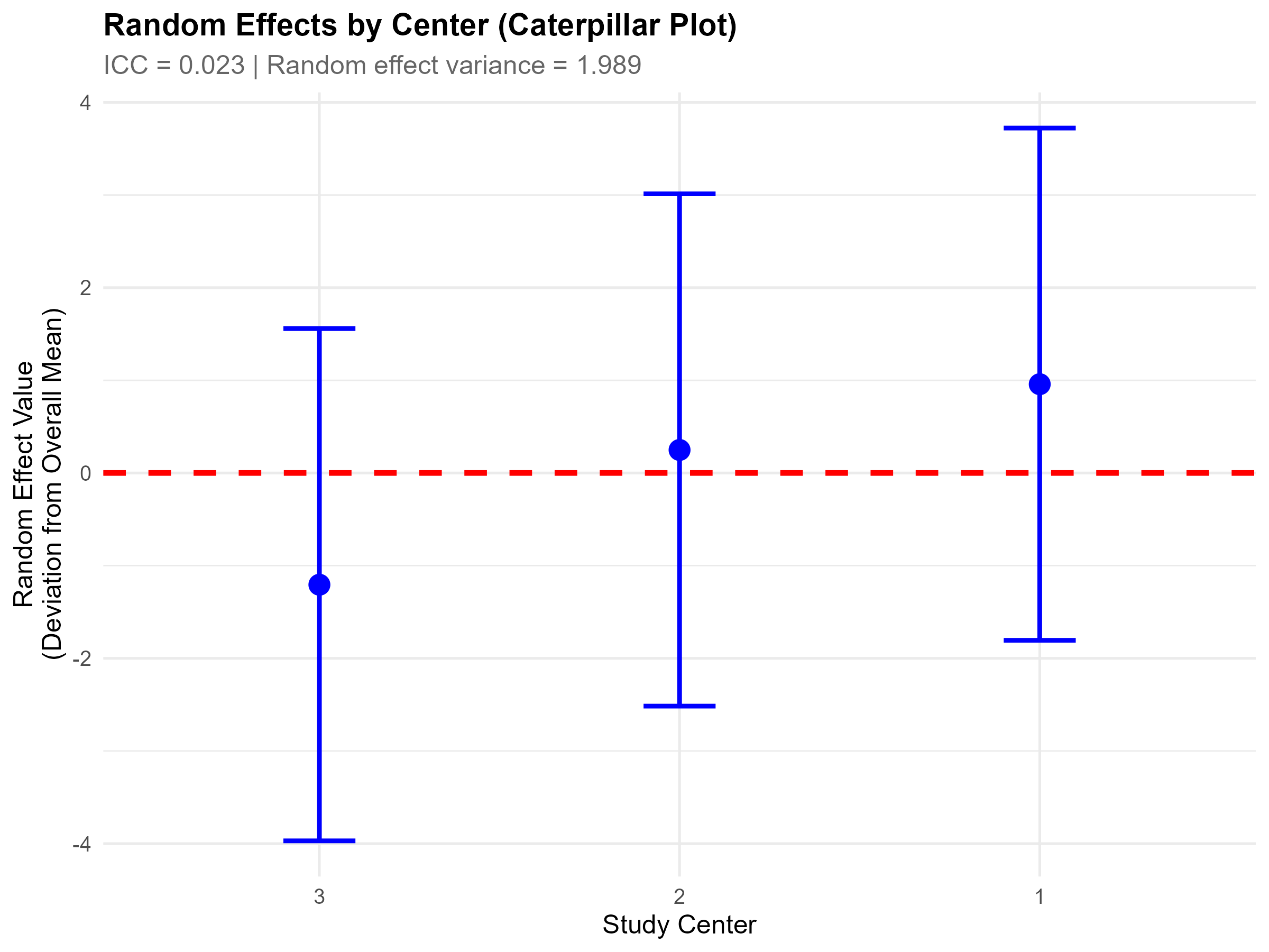


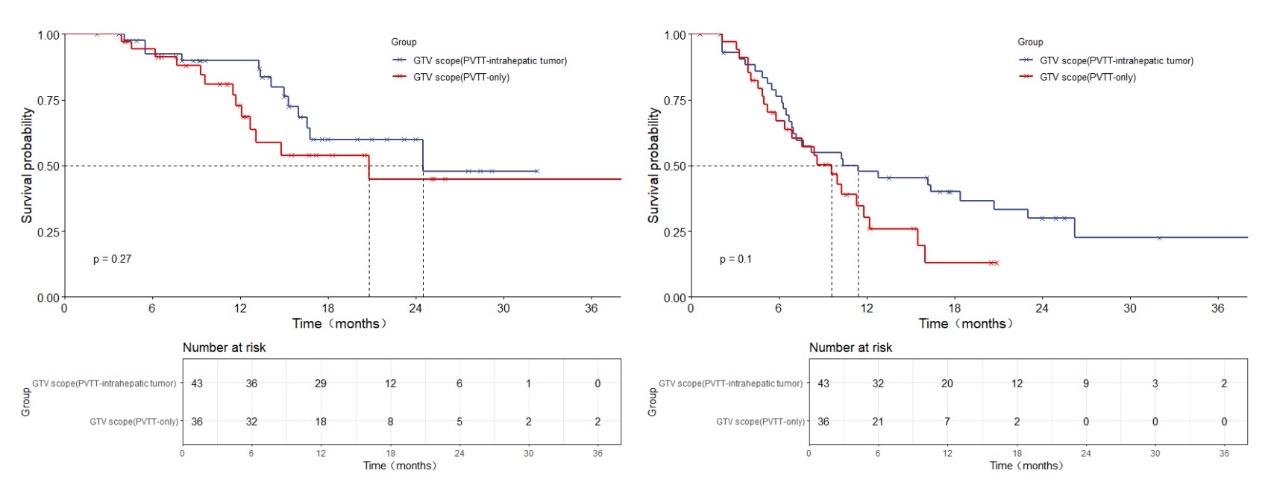


**Supplementary Figure1. Kaplan-Meier curves stratified by GTV scope in the HAIC-SBRT group.** (A) Comparison of Kaplan-Meier curves for overall survival (OS) between patients in the HAIC-SBRT group with "intrahepatic tumor plus PVTT" as the GTV scope and those with "PVTT only" as the GTV scope. (B) Comparison of Kaplan-Meier curves for progression-free survival (PFS) between patients in the HAIC-SBRT group with "intrahepatic tumor plus PVTT" as the GTV scope and those with "PVTT only" as the GTV scope.
